# Prediction of Recurrence-associated Death from Localized Prostate Cancer with a Charlson Comorbidity Index–reinforced Machine Learning Model

**DOI:** 10.1515/med-2019-0067

**Published:** 2019-08-14

**Authors:** Yi-Ting Lin, Michael Tian-Shyug Lee, Yen-Chun Huang, Chih-Kuang Liu, Yi-Tien Li, Mingchih Chen

**Affiliations:** 1 Department of Urology, St. Joseph Hospital, Yunlin County, 63241, Taiwan; 2 Graduate Institute of Business Administration, College of Management, Fu Jen Catholic University, New Taipei City 24205, Taiwan

## Abstract

Research has failed to resolve the dilemma experienced by localized prostate cancer patients who must choose between radical prostatectomy (RP) and external beam radiotherapy (RT). Because the Charlson Comorbidity Index (CCI) is a measurable factor that affects survival events, this research seeks to validate the potential of the CCI to improve the accuracy of various prediction models. Thus, we employed the Cox proportional hazard model and machine learning methods, including random forest (RF) and support vector machine (SVM), to model the data of medical records in the National Health Insurance Research Database (NHIRD). In total, 8581 individuals were enrolled, of whom 4879 had received RP and 3702 had received RT. Patients in the RT group were older and exhibited higher CCI scores and higher incidences of some CCI items. Moderate-to-severe liver disease, dementia, congestive heart failure, chronic pulmonary disease, and cerebrovascular disease all increase the risk of overall death in the Cox hazard model. The CCI-reinforced SVM and RF models are 85.18% and 81.76% accurate, respectively, whereas the SVM and RF models without the use of the CCI are relatively less accurate, at 75.81% and 74.83%, respectively. Therefore, CCI and some of its items are useful predictors of overall and prostate-cancer-specific survival and could constitute valuable features for machine-learning modeling.

## 1 Introduction

Localized prostate cancer is one of the most common male cancers and has led to unavoidable cancer death and impairment of quality of life [1, 2]. Current evidence has already demonstrated that great controversy exists regarding the treatment options for localized prostate cancer. Radical prostatectomy (RP) is associated with the lowest cancer-specific mortality in observational studies [3, 4], but selection bias exists. The selection bias means that patients with low comorbidity tend to undergo RP, whereas patients with high comorbidity are recommended to receive external beam radiotherapy (RT) [3, 4]. Randomized trials have demonstrated no significant differences between patients undergoing RP and patients undergoing RT in overall and cancer-specific survival. In addition, no differences are exhibited in long-term quality of life (QoL) for patients undergoing RP versus those undergoing RT [4]. Currently, the shared decision-making process has been made part of standard practice in medical decision management for localized prostate cancer. However, patients who must choose their treatments encounter a dilemma when they have information regarding only the reported statistical cancer outcomes and posttreatment QoL but are unable to take personal characteristics into consideration [3, 4]. Integrating cancer characteristics, comorbidity, and cancerous outcomes into a machine-learning model, and obtaining a predictive result might represent a solution to the dilemma.

Machine-learning modeling could be employed as a powerful tool for addressing the problem of treatment-related decisions for patients with prostate cancer. In the past 20 years, an increasing number of new, powerful algorithms and computer science advances have made the modeling of big medical data possible. The machine learning models formulated using big medical data enable individualized predictions of clinical outcomes. Several instances of this have already occurred in the areas of oncology outcome prediction. Algorithms, such as support vector machine (SVM), random forest (RF), artificial neural network, and decision tree algorithms, have been applied for modeling with acceptable accuracy [5]. Kourou et al. indicated that a small sample size represents the most common research limitation for applications of machine learning in past decades [5]. Furthermore, after reviewing the literature thoroughly, they also concluded that the dataset quality and careful feature selection are also crucial for effective machine learning and accurate prediction. Relatively few references currently utilize machine learning algorithms for prostate cancer research [6, 7]. Two studies have employed machine learning to maximize the detection accuracy of prostate cancer. The absence of research focusing on machine learning predictions of localized prostate cancer outcomes may be a result of the long survival times following treatment. The longer the duration of disease-free survival is, the greater the chances are that non-prostate-cancerous factors, such as comorbidity and accidents, constitute the primary determinants of survival. In addition to cancerous characteristics, therefore, this research investigates the effects of non-prostate-cancerous factors for modeling long-survival cancers.

The Charlson Comorbidity Index (CCI) [8] serves as a valuable, measurable indicator for improving prediction accuracy with machine learning models for long life expectancy cancers. The CCI has been recognized as influential in relation to the clinical outcomes for localized prostate cancer. Park et al. declared CCI to be a prognostic factor for RP outcomes [9]. Lee et al. demonstrated that CCI is a major prognostic factor for long-term survival after RP [10]. Later, Rajan et al. denied the effect of comorbidity on cancer-specific survival in prostate cancer patients [11]. However, they all found comorbidity to be a reliable predictor for overall survival. Comorbidity has also been proven to represent a strong predictor of overall survival for patients treated with radiotherapy. Jespersen CG et al. demonstrated that the choice of treatments for localized prostate cancer is affected by comorbidity [12]. Therefore, CCI is not only a factor that influences survival but is also an indicator for treatment options.

Taiwan’s National Health Insurance Research Database (NHIRD), created in 1998, is an excellent resource for medical research because of the compulsory enrollment involved in Taiwanese national health insurance. The medical expense records of more than 99% of Taiwan’s population are included in the NHIRD. The NHIRD currently provides 78 databases openly accessible, including 59 health databases, 5 social databases, and 14 welfare databases. The first to 14th database contents include health insurance–related data files relating to ambulatory patients, inpatients, pharmacy information and medical care orders, and cause-of-death data. As part of the NHIRD, the Taiwan Cancer Registry (TCR) was created by the Department of Health to collect cancer epidemiological data from 1996 onwards comprehensively. TCR has become an indispensable resource for Taiwan cancer research. Using the NHIRD, Wang’s work surveyed second primary malignancy risk after radiotherapy for patients with rectal cancer [13]. Researchers used the NHIRD to prove that the survival of lung cancer is affected by a common comorbidity, chronic renal insufficiency [14]. A type of Chinese herb was demonstrated to improve the survival of lung cancer with this database [15]. Wei et al. also used the NHIRD to clarify the effects of the comorbidity chronic kidney disease on the mortality risk of lung cancer patients [14]. Yang et al. recognized that CCI is a good predictor for NHIRD patients with lung cancer using a Cox regression model (16). Successively, fine results have been reported in NHIRD-related literature relating to breast cancer [17, 18] and hepatoma [19]. Prostate cancer–related issues with NHIRD have also been published [20, 21, 22]. However, these studies related to prostate cancer have not focused on cancerous recurrence or death. The big data of NHIRD have also been analyzed by various researchers using machine learning modeling. Hu et al. used the NHIRD to conduct machine learning predictions regarding return visits for pediatric patients to the emergency department [23]. Wang et al. utilized machine learning classifiers for the prediction of brain metastasis of lung cancer for patients from the NHIRD [24]. Some research has aimed to validate comorbidity data, such as acute myocardial infarction or stroke, and death data. High consistency among these data has been observed [25, 26, 27]. Thus, data derived from the NHIRD should be extremely suitable for the machine learning modeling of prostate cancer survival.

To our knowledge, no machine learning models have been able to predict localized prostate cancer outcomes. Although CCI has been proven to represent a significant prognostic factor for treatment outcome of localized prostate cancer, few studies have taken the CCI into account when building machine learning models. This study focused mainly on building a CCI-reinforced machine learning model for the prediction of different treatment outcomes. Thus, the purposes of this study were 1) to use data extracted from the NHIRD to compare the cancer outcomes for patients with localized prostate cancer who have undergone RP versus patients with localized prostate cancer who have undergone RT, 2) to analyze the factors that influence cancer-specific and overall survival, and 3) to observe the effects of the CCI on the improvement of prediction ability (and thus to determine whether the CCI represents a significant factor in cancer outcomes).

## 2 Materials and methods

### 2.1 Search for target population in database

**Figure 1:**
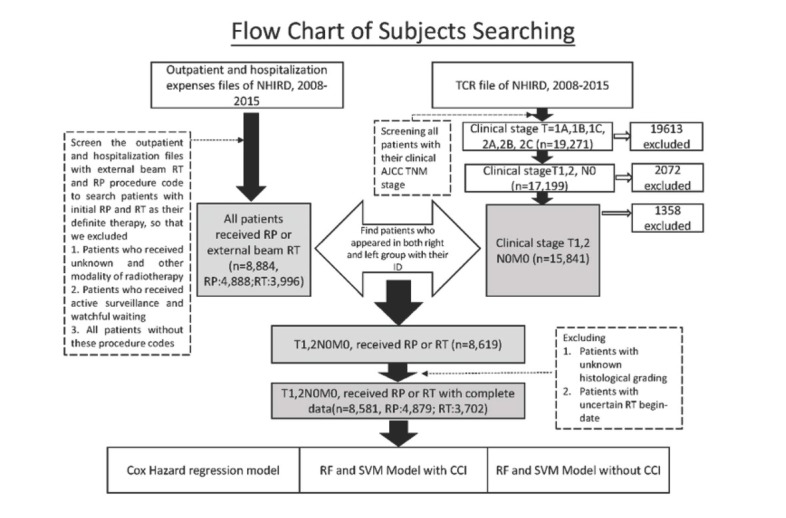
Flow chart of subjects searching This figure demonstrates whole procedure for establishing our target population. The dataset of target population was extracted from the outpatient expense file, hospitalization expense file, TCR file and death cause file of NHIRD.

This research was a retrospective study targeting patients with localized prostate cancer who had undergone RP or RT. Both ICD9 (185) and ICD-10 (C61) were used to identify patients with prostate cancer in the NHIRD from 2008 to 2015. To search for clinical T1N0M0, and T2N0M0 patients, the TCR database was incorporated to obtain information on the initial clinical stages of patients. The next step was using the radical prostatectomy procedure code (79403B, 79410B) and order codes for external beam radiation therapy (36015B, 36002B, 36005B, 36012B, 36019B) to search for patients who had received RP or RT as their initial definite treatment. Individuals with incomplete data on clinical stages, histological grading, or radiotherapy start-date, for example, were excluded. Patients whose recurrence statuses were recorded as “uncertain” or whose radiation modality was marked “unknown or other than external beam radiation therapy” in the TCR database were also excluded. The flow chart for the study population retrieved from NHIRD appears in Figure 1. The demographic statistical comparison between the RP group and the RT group is as seen in Table 1.

**Table 1:** Demographic features among treatment groups.

Variables		Different Treatment		
OP		RT	p-value	
No. (%) of patients	8581	4879	3702	
Age		65.79	74.1	<.0001*
T-stage	1A	88(1.80)	128(3.46)	<.0001*
	1B	96(1.97)	156(4.21)	
	1C	1512(30.99)	936(25.28)	
	2A	994(20.37)	617(16.67)	
	2B	580(11.89)	420(11.35)	
	2C	1609(32.98)	1445(39.03)	
Grade	1	481(9.86)	539(14.56)	<.0001*
	2	1903(39.00)	1361(36.76)	
	3	2495(51.14)	1802(48.68)	
Connective tissue disease		98(2.07)	63(1.70)	0.2995
Mild liver disease		166(3.40)	105(2.84)	0.158
Ulcer disease		839(17.20)	544(14.69)	0.0018*
Congestive heart failure		171(3.50)	214(5.78)	<.0001*
Peripheral vascular disease		117(2.40)	115(3.11)	0.0451*
Chronic pulmonary disease		629(12.89)	593(16.02)	<.0001*
Cerebrovascular disease		371(7.60)	410(11.08)	<.0001*
Diabetes		1079(22.12)	657(17.75)	<.0001*
Diabetes with end organ damage		4548(93.22)	3457(93.38)	0.7607
Moderate or severe renal disease		229(4.69)	199(5.38)	0.1507
Metastatic solid tumor		130(2.66)	51(1.38)	<.0001*
Hemiplegia		27(0.55)	38(1.03)	0.0123*
Solid tumor without metastasis		289(5.92)	300(8.10)	<.0001*
Myocardial infarct		44(0.90)	45(1.22)	0.1554
Dementia		47(0.96)	97(2.62)	<.0001*
Moderate or severe liver disease		3(0.06)	0(0)	0.1313
Any except malignancy, malignant including neoplasm lymphoma of skin and leukemia,		256(5.25)	270(7.29)	0.0003*
AIDS/HIV		1(0.02)	0(0)	0.8447
Overall mortality		88(1.80)	325(8.78)	<.0001*
F/U TIME		4.11	4.17	0.2517
Prostate cancer specific mortality		24(0.49)	79(2.13)	<.0001*
Patients with CCI Score equal to	0	2073(42.49)	1779(48.06)	<.0001*
	1	1346(27.59)	746(20.15)	
	2	571(11.70)	404(10.91)	
	3	321(6.58)	241(6.51)	
	4	203(4.16)	191(5.16)	
	5	127(2.60)	126(3.40)	
	6+	238(4.88)	215(5.81)	
CCI Average	^ ^	1.36	1.42	<.0001*

Grade 1: Gleason score 2~5; Grade 2: Gleason score 6,7; Grade 3: Gleason score 8~10

*: Statistically significant, p<0.05

### 2.2 Data collection and definition of variables

Various meaningful variables are extracted from the medical expense records and Cancer Registry records of every single patient in our target population. These variables could be categorized into cancerous characteristic variables, comorbidity variables, and cancerous outcome variables. Cancerous characteristic variables include age, clinical T stage, histological grade, initial definite treatment, and duration of follow-up. The cancer data are obtained from the TCR file. Comorbidity variables [28] are composited by recording the presence or absence of the following conditions, including myocardial infarction, congestive heart failure, peripheral vascular disease, cerebrovascular disease, dementia, chronic pulmonary disease, rheumatic disease, mild liver disease, diabetes without chronic complication, diabetes with chronic complication, hemiplegia or paraplegia, renal disease, any malignancy, including lymphoma and leukemia, except malignant neoplasm of skin, moderate or severe liver disease, metastatic solid tumor, AIDS/HIV, and the CCI. We obtained the status of every comorbidity item with ICD-9 code screening of every comorbidity contained in the CCI in the outpatient department and hospitalization expense database. The cancerous outcome variables, including overall survival time and cancer-specific survival time, were retrieved from the cause-of-death file. The aforementioned data were analyzed and modeled in the following sections.

The demographic and cancer characteristics are listed and compared in Table 1, such as comorbidities and cancerous outcomes among treatment groups extracted from the NHIRD. The chi-square test was used for categorical outcomes, and the independent *t* test was used for numeric outcomes.

### 2.3 Establish significant causal factors for cancerous outcomes with the Cox Hazard regression model

**Figure 2:**
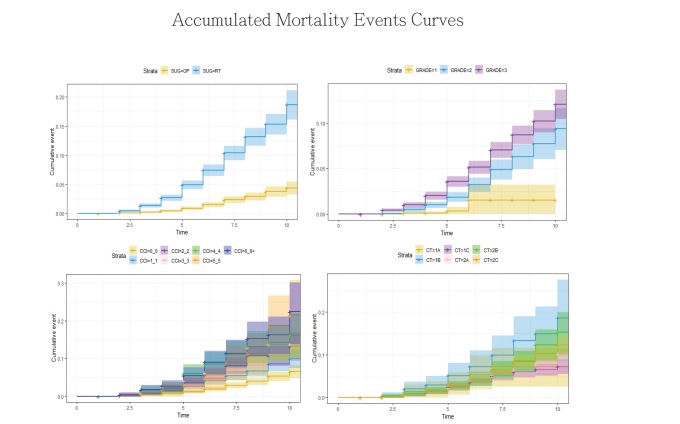
Accumulated mortality events curve, stratified initial definite treatment, grade, stage and years Mortality events are significantly higher in high grade, RT group. Grade 1: Gleason score 2~5; Grade 2: Gleason score 6,7; Grade 3: Gleason score 8~10 *: Statistically significant, p<0.05

We stratified all patients according to their T stage, grade, initial definite treatment, and CCI, and we compared the cancer-specific survival and overall survival using the Kaplan–Meier method (Figure 2). The significant causal factors were identified for recurrence-free survival, disease specific survival, and overall survival time using a Cox hazard regression model (Table 2).

**Table 2:** Hazard ratios of features in Cox proportional Hazard model

	Cox models											
Variables	Overall survival CCI only			Overall survival CCI item			Specific survival CCI only			Specific survival CCI item		

Cancerous Characteristic variables												
	HR	95%HR	p-value	HR	95%HR	p-value	HR	95%HR	p-value	HR	95%HR	p-value
Age	1.066	1.05-1.08	<.0001	1.058	1.04-1.07	<.0001	1.047	1.01-1.07	0.0023	1.04	1.01-1.07	0.0037
T-stage (ref. = 1A)												
1B	1.157	0.54 2.45	0.2798	1.239	0.58-2.63	0.5775	2.922	0.35-24.35	0.3216	1.775	0.23-1.24	0.2811
1C	0.697	0.36-1.34	0.3923	0.72	0.37-1.39	0.3296	1.829	0.24-13.53	0.5545	2.125	0.28-15.96	0.5756
2A	0.748	0.38-1.45	0.6994	0.785	0.40-1.53	0.4786	2.157	0.28-16.07	0.4532	2.032	0.26-15.56	0.4639
2B	0.875	0.44-1.77	0.4872	0.929	0.47-1.83	0.8314	2.118	0.27-16.11	0.4686	1.564	0.21-11.55	0.4949
2C	0.796	0.41-1.51	0.2798	0.833	0.43-1.59	0.5803	1.597	0.21-11.70	0.645	1.775		0.6614
Grade (ref. = 1)												
2	4.657	1.71-12.67	0.0026	4.496	1.65-12.24	0.0033	92..	0	0.9792	10..	0	0.9798
3	6.78	2.51-18.28	0.0002	6.463	2.39-17.44	0.0002	23..	0	0.9778	2...	0	0.9785
Different treatment (ref. = RT)	2.672	2.03-3.50	<.0001	2.693	2.04-3.54	<.0001	3.144	1.85-5.33	<.0001	3.194	1.88-5.41	<.0001
**Comorbidity items**												

CCI (ref. = 0)												
1	1.461	1.10-1.94	0.0088				0.79	0.46-1.34	0.3872			
2	1.816	1.32-2.49	0.0002				1.188	0.66-2.13	0.5634			
3	2.015	1.40-2.91	0.0002				0.868	0.38-1.96	0.7345			
4	2.455	1.68-3.59	<.0001				1.368	0.63-2.96	0.4263			
5	2.108	1.32-3.37	0.0018				0.486	0.11-2.02	0.3216			
6+	2.767	1.94-3.95	<.0001				1.864	0.96-3.60	0.0645			
Connective tissue disease				0.76	0.358-1.61	0.4761				0	0	0.9861
Mild liver disease				1.488	0.96-2.30	0.0757				1.95	0.84-4.49	0.1167
Ulcer disease				1.132	0.89-1.43	0.3052				1.13	0.69-1.84	0.6207
Congestive heart failure				2.172	1.63-2.88	<.0001				1.69	1.88-3.22	0.1107
Peripheral vascular disease				0.941	0.56-1.55	0.814				0.815	0.25-2.59	0.729
Chronic pulmonary disease				1.644	1.32-2.03	<.0001				1.18	0.73-1.89	0.4907
Cerebrovascular disease				1.5	1.05-1.73	0.019				1.197	0.69-2.05	0.5143
Diabetes				1.105	1.85-1.42	0.4354				0.774	0.44-1.34	0.361
Diabetes with end organ damage				1.092	0.75-1.57	0.6421				0.969	0.40-2.29	0.9429
Moderate or severe renal disease				1.35	0.96-1.9	0.0802				2.02	1.07-3.8	0.0283
Metastatic solid tumor				1.75	0.95-3.22	0.0718				3.87	1.65-9.09	0.0019
Hemiplegia				0.828	0.33-2.03	0.681						0.467
Solid tumor without metastasis				1.792	0.88-3.63	0.1058				0.734	0.10-5.33	0.7602
Myocardial infarct				0.805	0.35-1.83	0.6055				0.53	0.07-3.90	0.533
Dementia				1.874	1.26-2.78	0.0018				1.716	0.40-7.35	0.7527
Moderate or severe liver disease				36.78	0.96-1.90	0.0004				0	0	0.9988
Any malignancy, including lymphoma and leukemia, except malignant neoplasm of skin				0.692	0.32-1.47	0.3408				0.89	0.10-7.35	0.9142
AIDS/HIV				0	0	0.9706				0	0	0.9982

The RP and RT groups were pooled together. The factors which significantly affect the prostate-cancer-specific survival and overall survival are identified with Cox regression model.

Grade 1: Gleason score 2~5; Grade 2: Gleason score 6,7; Grade 3: Gleason score 8~10

*: Statistically significant, p<0.05

### 2.4 Comparison of machine learning models with and without CCI

Machine learning models, including RF and SVM, were adopted for the prediction of cancer outcomes. To prove that CCI would enhance the predictive ability of a machine learning model, we created the models using two stages. The first stage was training models with cancerous characteristic factors and follow-up durations as the inputs for the model and with cancerous outcomes as the outputs for the model. The only difference in the second stage was the addition of comorbidity variables along with the other input variables in stage one of the model, as seen in Table 3. The parameters for the setting of every algorithm were all by default. Three new data sets were constructed with the ratios of 1:1, 1:2, 1:3, to make up the imbalanced classes of cancer outcomes [29]. Five-fold cross validation was then applied to these three datasets. The mean accuracy, mean AUC, and mean kappa value feature in Table 4.

**Table 3:** List of variable used in machine learning modeling for overall survival prediction

	Machine learning models for overall survival			
Variables	RF With CCI	RF Without CCI	SVM With CCI	SVM Without CCI
**Cancerous Characteristic variables**				

Age (yr)	X	X	X	X
T-stage	X	X	X	X
Grade	X	X	X	X
Different treatment	X	X	X	X
Duration of follow up (yr)	X	X	X	X
**Comorbidity items**				
CCI	X		X	
Connective tissue disease	X		X	
Mild liver disease	X		X	
Ulcer disease	X		X	
Congestive heart failure	X		X	
Peripheral vascular disease	X		X	
Chronic pulmonary disease	X		X	
Cerebrovascular disease	X		X	
Diabetes	X		X	
Diabetes with end organ damage	X		X	
Moderate or severe renal disease	X		X	
Metastatic solid tumor	X		X	
Hemiplegia	X		X	
Solid tumor without metastasis	X		X	
Myocardial infarct	X		X	
Dementia	X		X	
Moderate or severe liver disease	X		X	
Any malignancy, including lymphoma and leukemia, except malignant neoplasm of skin	X		X	
AIDS/HIV	X		X	
Overall death	Y	Y	Y	Y

This table lists the feature selection during modeling. X represents variables which was put at the input site of machine learning model. Y represents variables put at the output site of machine learning model.

**Table 4:** Predictive ability of machine learning models

Overall death	RF			SVM		
	1:1	1:2	1:3	1:1	1:2	1:3
**With comorbidity as independent variables**						

Accuracy	0.8000	0.8095	0.8262	0.8518	0.7958	0.8116
Sensitivity	0.8500	0.6047	0.4198	0.8182	0.5213	0.3186
Specificity	0.7500	0.9157	0.9595	0.8133	0.9316	0.9777
Kappa	0.6000	0.5512	0.4480	0.6315	0.4955	0.3724
**Without comorbidity as independent variables**						

Accuracy	0.7483	0.7324	0.8024	0.7527	0.7621	0.7837
Sensitivity	0.7027	0.5571	0.3690	0.7660	0.5312	0.1971
Specificity	0.7945	0.8182	0.9451	0.7391	0.8763	0.9810
Kappa	0.4969	0.3823	0.3721	0.5052	0.4314	0.2369

The CCI- reinforced and CCI absent RF and SVM models were built. We evaluated the ability of model with accuracy, sensitivity, specificity and kappa.

### 2.5 Software

SAS version 9.4 was used for data extraction, data preprocessing from the NHIRD, and Cox regression analysis. The R studio 3.5.1 was used as a platform to implement the machine learning models. Several R packages were used, including randomForest for RF modeling, e1071 for SVM modeling, pROC and caret for calculating accuracy and kappa, and survival and survminer for Kaplan–Meier.

## 3 Results

In total, 8581 patient’s records were included in the study: 4879 who had received radical prostatectomies (the RP group) and 3702 who had received radiotherapy (the RT group). As seen in Table 1, RP group members received fewer diagnoses of congestive heart failure (171(3.50%) in RP vs 214(5.78%) in RT, p < 0.0001), peripheral vascular disease (117(2.40) vs 115(3.11), p = 0.0451), chronic pulmonary disease (629(12.89) vs 593(16.02), p < 0.001), cerebrovascular disease (371(7.60) vs 410(11.08), p < 0.0001), hemiplegia (27(0.55) vs 38(1.03), p = 0.0123), and dementia (47(0.96) vs 97(2.62), p < 0.0001), any malignancy (256(5.25) vs 270(7.29)) as well as lower CCI scores (1.36 vs 1.42, p < 0.0001), less overall death (88(1.80) vs 325(8.78), p < 0.0001), and prostate cancer–specific death (24(0.49) vs 79(2.13), p < 0.0001). However, exceptions also occurred. We observed that more patients had ICD-9 codes for ulcer disease (839(17.20) vs 544(14.69)), diabetes mellitus without complications (1079(22.12) vs 657(17.75), p < 0.0001), and metastasis solid tumor (130(2.66) vs 51(1.38), p < 0.0001) in the RP group. Our data also revealed that the RP and RT groups included equal incidences of any severity of liver disease, diabetes mellitus with complications, ulcer disease, connective tissue disease, and follow-up time.

The Cox proportional hazard model was established, as shown in Table 2. The CCI was stratified into seven levels equivalent to CCIs of 0, 1, 2, 3, 4, 5, and 6 or more. Compared with patients with CCI0, Patients with CCI1, CCI2, CCI3, CCI4, CCI5, and CCI6+ had statistically significantly higher risk of overall death, with the hazard ratios 1.461, 1.816, 2.015, 2.455, 2.108, and 2.767. Grade, initial definite treatment, and age also played significant roles in the risk of overall death. Among CCI items, moderate-to-severe liver disease together with dementia, congestive heart failure, chronic pulmonary disease, and cerebrovascular disease were significantly associated with higher overall risk of death. Conversely, the patients classified as CCI1, CCI2, CCI3, CCI4, CCI5, and CCI6+ had statistically nonsignificant risks for prostate cancer-specific death compared with patients classified as CCI0. Grade and initial definite treatment were significantly associated with higher risk of prostate cancer–specific mortality. Metastatic solid tumors followed by moderate-to-severe renal disease would increase the risk of prostate cancer–specific death. The accumulated survival event curves stratified with age, initial definite treatment, grade, and CCI were plotted. The Kaplan–Meier test revealed that the RP group, low grade groups, and low CCI groups were statistically significantly associated with fewer overall mortality events (Figure 2).

Because the CCI and its items were highly correlated with the overall mortality rather than with prostate cancer–specific mortality, we created models with and without CCI and the related items to predict the overall numbers of deaths instead of prostate cancer–specific deaths. The overall mortality in our dataset was 413, so we had to downsample the relatively larger group of patients who survived until the end of the follow-up period with the numbers 413, 826, and 1239 according to the previously designed ratios of 1:1, 1:2, and 1:3. The CCI-reinforced SVM model with a 1:1 sampling ratio yielded the best accuracy and kappa (0.8518, 0.6315) for prediction of overall numbers of deaths. SVM without CCI yielded a less accurate model with a smaller kappa value (0.7581, 0.5164). The same phenomenon was observed in the case of an RF model. The CCI-reinforced RF model had higher accuracy and kappa (0.80, 0.60) than the RF model without CCI had (0.7483, 0.4946). Different downsampling ratios for the survival groups did not affect the accuracy or kappa significantly, as ratios of 1:1, 1:2, and 1:3 selected. (Table 4)

## 4 Discussion

Patient selection for RP or RT is an art for urologists and patients. According to the popular clinical guidelines followed by most urologists in Taiwan, flow charts in NCCN clinical practice guidelines for prostate cancer 2010 [30] recommended that RPs are more suitable for localized prostate cancer patients with life expectancies of more than 10 years. This is because risks associated with RP are higher than those for radiotherapy during the procedure. RP also yields a better cancerous outcome in intermediateand high-risk localized prostate cancer than RT does. For patients with life expectancies of less than 10 years and with lower clinical T stages and histological grading, RT might be sufficient to protect them from mortality caused by prostate cancer. In Taiwan, the cutoff age for a 10-year life expectancy is 76 years during the study period. Average ages (RP: 65.79 years old, RT: 74.10) for these two groups in our dataset are consistent with the NCCN recommendation, as seen in Table 1. More young patients personally chose or were advised by physicians to undergo radical prostatectomies. In addition, we found that patients with fewer comorbidities tended to undergo radical prostatectomies rather than radiotherapy (Table 1). This finding is compatible with Jespersen’s work, in which the choice of treatment for localized prostate cancer is demonstrated to be affected by comorbidity [12]. This is relatively reasonable because RP is a major surgery, and its success is highly dependent on patients’ general conditions. Systemic diseases yield more perioperative complications and prolong hospital stay [31]. Our findings furthermore revealed that the RT group had less favorable prostate cancer–specific mortality. This finding is consistent with Abdollah’s work and the subsequent systemic review and meta-analysis. This might result from the nature of sparing radiation insensitive tumor cells during radiotherapy, which would not happen in the case of RP [32, 33]. In the RP group in our dataset, more patients had diabetes mellitus without end organ damage compared with other comorbidities. This may have been due to routine blood sugar tests according to clinical practice guidelines for preoperative blood tests [34]. Routine blood glucose checkup led to exhaustive detection and even overdiagnosis of diabetes mellitus.

Our data from the NHIRD of Taiwan revealed that CCI and associated items are critical risk factors for the overall survival of localized prostate cancer patients but not for prostate cancer–specific survival. These findings are consistent with the relevant literature. Matthes et al. surveyed the effects of comorbidities for prostate cancer victims. They found that CCI (CCI1: HR: 2.07 (1.51-2.85) and CCI2+: 2.34 (1.59-2.34)) were associated with overall mortality rather than prostate cancer–specific mortality [35]. The increase in the overall mortality risk was also aligned with our results. Thomas’ study demonstrated the same conclusions in patients receiving RP [36]. The findings of Jae et al. also supported this assertion in patients with RP [37]. In line with our results, Matthes claimed that CCI played a more significant role than age in overall mortality risks [38]. Cancela and colleagues presented contradictory findings that age remains a major predictor in the untreated localized prostate cancer population [39]. To our knowledge, much literature addresses the measurement of the risks for every single CCI item. We established a Cox proportional hazard model to assess the effects of all CCI items and observed severe liver disease and dementia to be the most important factors associated with overall mortality. Minor factors, including congestive heart failure, chronic pulmonary disease, cerebrovascular disease, were also discovered. Because the score for moderate-to-severe liver disease is 3, moderate-to-severe liver disease has a higher correlation with overall mortality. Chen et al. studied the population for individuals with dementia in Taiwan and found them to usually have multiple comorbidities and to be poorly cared for [40]. This might be the reason why dementia is relevant. In clinical practice, informing patients and their families to take dementia into consideration during their decision-making processes might be advisable.

Our data suggested that the CCI and its items can reinforce the machine learning classifier for survival prediction of localized prostate cancer. As a good predictor of overall death, CCI and its items could be extracted from the NHIRD and successfully modeled with RF and SVM algorithms. The highest accuracy rate of our model is 85.18%, with the kappa value 0.6315. Fleiss defined the quality of classifier as good to fair with kappa values between 0.4 and 0.75 [41]. Currently, researchers only relatively rarely model prostate cancer survival with machine learning algorithms. This study might constitute a useful reference for other research. Imbalanced classification is fairly common in most real-world cases, and our dataset used a ratio of 1:20. Downsampling is a well-known and widely used technique for solving imbalance problems [29]. In this study, we followed the recommendations of this handbook that the ratios of 1:1, 1:2, and 1:3 be used, and we observed that accuracy did not change significantly. However, the kappa value and sensitivity decreased as the ratio of sampling increased from 1:1 to 1:3. This occurred because the false negative prediction increased as the ratio of sampling increased. Subsequently, even the 1:3 RF model with CCI yielded a maximum accuracy of up to 82.62% among RF models, but we still regarded the 1:1 RF model with CCI as the best model among RF models because it has both high accuracy and kappa. Our dataset suggested that SVM models with CCI were superior to RF models with CCI. However, we still cannot conclude with certainty that SVM is a superior algorithm to RF. The advantage of RF is that RF is more interpretable because the RF algorithm enables the measuring of variables’ importance. This is helpful for variable selection and improved modeling.

In this research, we successfully identified the risk factors for prostate cancer death and all deaths among patients with localized prostate cancer in Taiwan. The research findings could serve as a valuable reference for epidemiological research in the east Asian prostate cancer population, whose clinical courses and epidemiological features differ from Caucasian and Black populations’ [42, 43, 44]. The model we established could be helpful clinically during the shared decision-making process. The nature of compulsory enrollment in Taiwan’s National Health Insurance enabled the comprehensive compilation of medical expense records and ensured that NHIRD constitutes a high-quality database in terms of data integrity. Integrity is an essential property for a database to assure consistency and statistical accuracy [45]. To maintain dataset integrity, we strive to avoid unnecessary sample dropouts. Therefore, a new method was designed to model our dataset with RF and SVM algorithms.

Because follow-up time can affect the duration of survival event observation, the follow-up time was included among the inputs for machine learning models. We have validated this new method of machine learning modeling for survival prediction using our dataset for localized prostate cancer, a cancer well-known to be associated with long survival. The longer the expected survival is, the longer follow-up time is required for observing survival events. Numerous researchers use fixed follow-up times for some cancers with shorter target event-free survival, for example, 3 years or 5 years [46, 47, 48, 49, 50]. However, no definite conclusions may be drawn regarding what follow-up time for observation of localized prostate cancer survival is adequate: this might be 10 years, 20 years, or even longer. Such follow-up times are too long for most databases to accomplish complete studies, currently. Using this right-censoring method, patients who have inadequate follow-up time can be retained while compared with the fixed follow-up time, such as 3 or 5 years, for observing survival events. Retaining the information provided by the inadequate follow-up records is helpful to maintain the integrity of our database for facilitating unbiased analysis and interpretation. In this study, we proved the method used to be feasible. The accuracy of our machine learning model was up to 80%. In clinical practice, we can assign follow-up times (t) as 1 year, 2 years, and so on to predict survival.

According to our findings in this study, we strongly suggest that researchers take CCI and associated items into account when developing new models for prostate cancer decision aids in the future. Furthermore, our machine learning models with satisfying accuracy, can serve for a decision aid during shared decision-making process [51]. It is helpful for physicians and patients to use our model to predict the survivals under different choices of definite treatment for prostate cancer. However, we cannot make suggestions until further evaluation of patients’ psychological impacts after their receiving of such machine-learning suggestions. Before our comorbidity-reinforced model could be added into standard shared decision-making process [51] in clinical practice, we suggest that trials for assessment of patients’ psychological and emotional impacts caused by machine learning suggestions need to be conducted. After these results are available, we might be able to design a new shared decision-making process reinforced by machine-learning decision aid..

This research has three limitations. First, prostate specific antigen (PSA) data is unavailable in the NHIRD. The pretreatment PSA level and the elevation of the PSA level after definite treatment of localized prostate cancer is associated with higher risks of prostate cancer–specific death [52]. Williams et al. also demonstrated that both pretreatment and posttreatment PSA levels were associated with an overall mortality of localized prostate cancer [53]. The lack of data regarding PSA would represent a major loss of crucial information for the status of extension of prostate cancer and would compromise the establishment of the Cox hazard proportional model and machine learning models. However, little evidence addresses the association between the PSA level and noncancerous mortality of prostate cancer. In our dataset, the proportion of non-cancerous mortality is as high as 75.06% of the overall mortality. The high proportion of noncancerous death in our dataset would attenuate the effects of the PSA data deficiency. Furthermore, our machine learning model achieves accuracy of up to 81 %, even in the absence of PSA data. This is an excellent proof of the significant role of CCI and its items.

The second limitation is that no uniform procedure was available for surveying the patients’ comorbidities, which often exist in retrospective datasets. The lack of standard procedures resulted in the neglect of some preexisting comorbidities and the underestimating of CCI. However, this bias can be partially diminished by routine preoperative evaluation, which is commonly performed in most Taiwanese medical centers and provides indications regarding some occult comorbidities. Although many machine learning models exist for prediction, they have seldom been used in clinical practice [54]. Future studies might aim at surveying the effects of various machine learning models in clinical practice.

The third limitation is the lack of records regarding self-paid treatments in the NHIRD database. Overcoming this limitation is difficult. Some brachytherapy, intensity-modulated radiotherapy, image-guided radiotherapy, robotic surgery, or laparoscopic RP are partially or fully paid for by patients themselves. The NHIRD database provides no information regarding the exact methods for the RT or RPs that patients undergo. This limitation causes bias for model building and survival prediction. However, National Health Insurance has begun to implement the policy of recording the code for every self-paid treatment. The respective problem will no longer exist in the future.

## 5 Conclusions

The CCI and its items are powerful predictors for cancerous outcomes. Using the CCI and its items for building machine learning models would enhance the predictive power of machine learning models utilizing RF and SVM algorithms. The various treatment choices are statistically insignificant after adjustment by Cox regression to cancerous outcomes. The outcome prediction of comorbidity-reinforced machine learning models has achieved acceptably high accuracy and thus could serve as an individualized decision aid during the shared medical decision-making process for localized prostate cancer after evaluation of psychological and emotional impacts caused by suggestions yielded with the model.

## Abbreviations

RP: radical prostatectomy
RT: external beam radiotherapy
CCI: Charlson comorbidity index
RF: random forest
SVM: support vector machine
NHIRD: National Health Insurance Database
QoL: quality of life
TCR: Taiwan Cancer Registry

## References

[1] Lehto US, Ojanen M, Vakeva A, Dyba T, Aromaa A, Kellokumpu-Lehtinen P (2019). Early quality-of-life and psychological predictors of disease-free time and survival in localized prostate cancer. Qual Life Res.

[2] Adam S, Feller A, Rohmman S, Arndt V (2018). Health-related quality of life among long-term (≥5 years) prostate cancer survivors by primary intervention: a systematic review. Health Qual Life Outcomes.

[3] Serrell EC, Pitts D, Hayn M, Beaule L, Hansen MH, Sammon JD (2018). Review of the comparative effectiveness of radical prostatectomy, radiation therapy, or expectant management of localized prostate cancer in registry data. Urol Oncol.

[4] Wallis CJ, Glaser A, Hu JC, Huland H, Lawrentschuk N, Moon D (2018). Survival and complications following surgery and radiation for localized prostate cancer: an international collaborative review. European urology.

[5] Kourou K, Exarchos TP, Exarchos KP, Karamouzis MV, Fotiadis DI (2015). Machine learning applications in cancer prognosis and prediction. Computational and structural biotechnology journal.

[6] Alkadi R, Taher F, El-baz A, Werghi NJ (2018). A Deep Learning-Based Approach for the Detection and Localization of Prostate Cancer in T2 Magnetic Resonance Images J Digit Imaging.

[7] Wang G, Teoh JY-C, Choi K-S (2018). Diagnosis of prostate cancer in a Chinese population by using machine learning methods. Conf Proc IEEE, Eng Med Biol Soc.

[8] Charlson ME, Pompei P, Ales KL, MacKenzie CR (1987). A new method of classifying prognostic comorbidity in longitudinal studies: development and validation. Journal of Chronic Disease.

[9] Koppie TM, Serio AM, Vickers AJ, Vora K, Dalbagni G, Donat SM (2008). Age‐adjusted Charlson comorbidity score is associated with treatment decisions and clinical outcomes for patients undergoing radical cystectomy for bladder cancer. Cancer: Interdisciplinary International Journal of the American Cancer Society.

[10] Lee JY, Lee DH, Cho NH, Rha KH, Choi YD, Hong SJ (2014). Charlson comorbidity index is an important prognostic factor for long-term survival outcomes in Korean men with prostate cancer after radical prostatectomy. Yonsei medical journal.

[11] Rajan P, Sooriakumaran P, Nyberg T, Akre O, Carlsson S, Egevad L (2017). Effect of Comorbidity on Prostate Cancer– Specific Mortality: A Prospective Observational Study. Journal of Clinical Oncology.

[12] Jespersen CG, Nørgaard M, Jacobsen JB, Borre MJ (2015). Patient comorbidity is associated with conservative treatment of localized prostate cancer. Scandinavian journal of urology.

[13] Wang T-H, Liu C-J, Chao T-F, Chen T-J, Hu Y-W (2018). Second primary malignancy risk after radiotherapy in rectal cancer survivors. World journal of gastroenterology.

[14] Wei Y-F, Chen J-Y, Lee H-S, Wu J-T, Hsu C-K, Hsu Y-C (2018). Association of chronic kidney disease with mortality risk in patients with lung cancer: a nationwide Taiwan population-based cohort study.

[15] Wang C-Y, Huang H-S, Su Y-C, Tu C-Y, Hsia T-C, Huang S-T (2018). Conventional treatment integrated with Chinese herbal medicine improves the survival rate of patients with advanced non-small cell lung cancer. Complementary therapies in medicine.

[16] Yang C-C, Fong Y, Lin L-C, Que J, Ting W-C, Chang C-L (2017). The age-adjusted Charlson comorbidity index is a better predictor of survival in operated lung cancer patients than the Charlson and Elixhauser comorbidity indices. European Journal of Cardio-Thoracic Surgery.

[17] Ding D-C, Chen W, Wang J-H, Lin S-Z (2018). Association between polycystic ovarian syndrome and endometrial, ovarian, and breast cancer: A population-based cohort study in Taiwan. Medicine.

[18] Hung SC, Liao KF, Hung HC, Lin CL, Lee PC, Hung SJ (2019). Tamoxifen use correlates with increased risk of hip fractures in older women with breast cancer: A case–control study in Taiwan. Geriatr Gerontol Int.

[19] Tsai W-C, Kung P-T, Wang Y-H, Kuo W-Y, Li Y-HJPo (2018). Influence of the time interval from diagnosis to treatment on survival for early-stage liver cancer. PloS one.

[20] Kao HH, Kao LT, Li IH, Pan KT, Shih JH, Chou YC (2019). Androgen Deprivation Therapy Use Increases the Risk of Heart Failure in Patients With Prostate Cancer: A Population‐ Based Cohort Study. J Clin Pharmacol.

[21] Kao LT, Xirasagar S, Lin HC, Huang CY (2019). Association Between Pioglitazone Use and Prostate Cancer: A Population‐Based Case‐Control Study in the Han Population. J Clin Pharmacol.

[22] Jhan J-H, Yeh H-C, Chang Y-H, Guu S-J, Wu W-J, Chou Y-H (2018). New-onset diabetes after androgen-deprivation therapy for prostate cancer: A nationwide propensity score-matched four-year longitudinal cohort study. J Diabetes Complications.

[23] Hu Y-H, Tai C-T, Chen SC-C, Lee H-W, Sung S-F (2017). biomedicine pi. Predicting return visits to the emergency department for pediatric patients: Applying supervised learning techniques to the Taiwan National Health Insurance Research Database. Computer methods and programs in biomedicine.

[24] Wang K-J, Adrian AM, Chen K-H, Wang K-M (2015). biomedicine pi. A hybrid classifier combining borderline-SMOTE with AIRS algorithm for estimating brain metastasis from lung cancer: A case study in taiwan. Computer methods and programs in biomedicine.

[25] Cheng CL, Lee CH, Chen PS, Li YH, Lin SJ, Yang YH (2014). Validation of acute myocardial infarction cases in the national health insurance research database in taiwan. J Epidemiol.

[26] Cheng CL, Chien HC, Lee CH, Lin SJ, Yang YH (2015). Validity of in-hospital mortality data among patients with acute myocardial infarction or stroke in National Health Insurance Research Database in Taiwan. Int J Cardiol.

[27] Cheng CL, Kao YH, Lin SJ, Lee CH, Lai ML (2011). Validation of the National Health Insurance Research Database with ischemic stroke cases in Taiwan. Pharmacoepidemiol Drug Saf.

[28] Charlson ME, Pompei P, Ales KL, MacKenzie CR (1987). A new method of classifying prognostic comorbidity in longitudinal studies: development and validation. Journal of chronic diseases.

[29] Chawla NV (2010). Data mining for imbalanced datasets: An overview. Data mining and knowledge discovery handbook: Springer;.

[30] Mohler JL (2010). The 2010 NCCN clinical practice guidelines in oncology on prostate cancer.

[31] Potretzke AM, Kim EH, Knight BA, Anderson BG, Park AM, Figenshau RS (2016). Patient comorbidity predicts hospital length of stay after robot-assisted prostatectomy. Journal of robotic surgery.

[32] Abdollah F, Schmitges J, Sun M, Jeldres C, Tian Z, Briganti A (2012). Comparison of mortality outcomes after radical prostatectomy versus radiotherapy in patients with localized prostate cancer: a population‐based analysis. International Journal of Urology.

[33] Petrelli F, Vavassori I, Coinu A, Borgonovo K, Sarti E, Barni S (2014). Radical prostatectomy or radiotherapy in high-risk prostate cancer: a systematic review and metaanalysis. Clin Genitourin Cancer.

[34] Guidance N (2018). Routine preoperative tests for elective surgery. BJU International.

[35] Matthes KL, Limam M, Pestoni G, Held L, Korol D, Rohrmann S (2018). Impact of comorbidities at diagnosis on prostate cancer treatment and survival. Journal of cancer research and clinical oncology.

[36] Guzzo TJ, Dluzniewski P, Orosco R, Platz EA, Partin AW, Han MJ (2010). Prediction of mortality after radical prostatectomy by Charlson comorbidity index. Urology.

[37] Koppie TM, Serio AM, Vickers AJ, Vora K, Dalbagni G, Donat SM (2008). Age‐adjusted Charlson comorbidity score is associated with treatment decisions and clinical outcomes for patients undergoing radical cystectomy for bladder cancer.

[38] Matthes KL, Limam M, Pestoni G, Held L, Korol D, Rohrmann S (2018). Impact of comorbidities at diagnosis on prostate cancer treatment and survival.

[39] de Camargo Cancela M, Comber H, Sharp L (2013). Age remains the major predictor of curative treatment non-receipt for localised prostate cancer: a population-based study. British journal of cance.

[40] Chen T-B, Yiao S-Y, Sun Y, Lee H-J, Yang S-C, Chiu M-J (2017). Comorbidity and dementia: a nationwide survey in Taiwan. PLoS One.

[41] Fleiss J (1981). Statistical methods for rates and proportions 2nd edition1981.

[42] Chao GF, Krishna N, Aizer AA, Dalela D, Hanske J, Li H (2016). Asian Americans and prostate cancer: A nationwide population-based analysis. Urol Oncol.

[43] Jeong IG, Dajani D, Verghese M, Hwang J, Cho YM, Hong JH (2016). Differences in the aggressiveness of prostate cancer among Korean, Caucasian, and African American men: A retrospective cohort study of radical prostatectomy. Urol Oncol.

[44] Taitt H (2018). Global Trends and Prostate Cancer: A Review of Incidence, Detection, and Mortality as Influenced by Race, Ethnicity, and Geographic Location. American journal of men’s health.

[45] Ravetz J (2018). Integrity must underpin quality of statistics. Nature.

[46] Facciorusso A, Di Maso M, Serviddio G, Vendemiale G, Spada C, Costamagna G (2016). Factors associated with recurrence of advanced colorectal adenoma after endoscopic resection. Clinical Gastroenterology and Hepatology.

[47] Kim W, Kim KS, Park RW (2016). Nomogram of Naive Bayesian Model for Recurrence Prediction of Breast Cancer. Healthcare informatics research.

[48] Ramkumar C, Buturovic L, Malpani S, Kumar Attuluri A, Basavaraj C, Prakash C (2018). Development of a Novel Proteomic Risk-Classifier for Prognostication of Patients With Early-Stage Hormone Receptor–Positive Breast Cancer. Biomarker insights.

[49] Shinagare AB, Balthazar P, Ip IK, Lacson R, Liu J, Ramaiya N (2018). High-Grade Serous Ovarian Cancer: Use of Machine Learning to Predict Abdominopelvic Recurrence on CT on the Basis of Serial Cancer Antigen 125 Levels. J Am Coll Radiol.

[50] Takada M, Sugimoto M, Masuda N, Iwata H, Kuroi K, Yamashiro H (2018). Prediction of postoperative disease-free survival and brain metastasis for HER2-positive breast cancer patients treated with neoadjuvant chemotherapy plus trastuzumab using a machine learning algorithm. Breast cancer research and treatment.

[51] Elwyn G, O’Connor A, Stacey D, Volk R, Edwards A, Coulter A (2006). The International Patient Decision Aid Standards (IPDAS) Collaboration. Developing a quality criteria framework for patient decision aids: online international Delphi consensus process. British Medical Journal.

[52] Van den Broeck T, van den Bergh RC, Arfi N, Gross T, Moris L, Briers E (2019). Prognostic Value of Biochemical Recurrence Following Treatment with Curative Intent for Prostate Cancer: A Systematic Review. European urology.

[53] Williams SG, Duchesne GM, Millar JL, Pratt GR (2004). Both pretreatment prostate-specific antigen level and posttreatment biochemical failure are independent predictors of overall survival after radiotherapy for prostate cancer. International Journal of Radiation Oncology, Biology, Physics.

[54] Kourou K, Exarchos TP, Exarchos KP, Karamouzis MV, Fotiadis DI (2015). Machine learning applications in cancer prognosis and prediction.

